# Methods to identify linear network models: a review

**DOI:** 10.1186/s41937-017-0011-x

**Published:** 2018-02-05

**Authors:** Arun Advani, Bansi Malde

**Affiliations:** 1 University of Warwick, Coventry and Institute for Fiscal Studies, London, UK; 2 University of Kent, Canterbury and Institute for Fiscal Studies, London, UK

**Keywords:** Networks, Social effects, Peer effects, Econometrics, C31, C81, Z13

## Abstract

In many contexts we may be interested in understanding whether direct connections between agents, such as declared friendships in a classroom or family links in a rural village, affect their outcomes. In this paper, we review the literature studying econometric methods for the analysis of linear models of *social effects*, a class that includes the ‘linear-in-means’ local average model, the local aggregate model, and models where network statistics affect outcomes. We provide an overview of the underlying theoretical models, before discussing conditions for identification using observational and experimental/quasi-experimental data.

## Background

Researchers and policymakers are often interested in identifying whether and the extent to which direct connections between agents affect their outcomes. For example, does the schooling performance of an individual depend on that of her friends? Does the health seeking behaviour of one’s relatives influence one’s own health seeking behaviour? Are firms’ investment and pay decisions influenced by the behaviour of firms in the same or closely-related industry? The identification and estimation of such social or network effects—direct spillovers from the characteristics or outcomes of one agent to the outcome of others—is of central interest in empirical research on networks in economics.

This paper reviews recent developments in methods to identify linear peer effect models using networks data—data with detailed information on the exact interactions between agents—when a single cross section of data is available. Linear models are the most widely used in empirical work, with many econometric methods developed to work with these, making them a natural choice to consider in this review^1^. Moreover, panel data on the network have until recently only rarely been available, so the majority of methods focus on the case with a single cross section of data^2^.

We provide an overview of a number of commonly used empirical specifications, the underlying theoretical models that generate them; and the conditions for the causal identification of parameters with cross-sectional data. We first consider three ‘local’ models, where only an agent’s direct connections (or *neighbours*) affect his outcome. The three specifications allow this effect to depend on the average outcome, total outcome, or both, of his neighbours. In the absence of information on interactions within a network (or group), identification of social effect parameters is greatly complicated by the so-called reflection problem, a form of simultaneity where it is not possible to identify who influences whom within the network or reference group (Manski 1993). Information on the exact interactions within a network can break this simultaneity for a wide range of network structures, allowing for identification of social effect parameters^3^.

Recent theoretical analyses have shown that the structure of networks, as well as positions of agents within them influences agents’ overall outcomes on dimensions such as information diffusion and risk sharing, among others (Bloch et al. 2008; Jackson et al. 2012; Banerjee et al. 2013). The availability of detailed network data has motivated the testing of implications of these models in recent work. We thus next discuss models where the entire structure of the network might matter for an individual’s outcome. Finally, we discuss how experimental and quasi-experimental variation could be used to provide additional variation to uncover social effects.

Our objective is to provide an overview of methods developed under the assumption that the network is (conditionally) exogenously formed: that is, there are no unobserved individual variables within the network that determine who links with whom. This is an important issue that is the subject of much recent research. A companion paper (Advani and Malde 2016), as well as other recent reviews (e.g. Graham 2015; Chandrasekhar 2015; de Paula 2016), provide overviews of this issue and of possible methods to deal with this.

An important issue in the practical estimation of social effects relates to the definition and measurement of the network. Our review proceeds assuming that the researcher perfectly observes and measures the network (or network neighbours) relevant for the outcome(s) of interest. Clearly, the researcher’s choice of network to use will influence the estimated parameters and potentially lead to different policy implications. However, few existing datasets collect information on more than one type of network, so that in practice, researchers are often restricted in their definition of the network by the available data. Nonetheless, existing studies indicate that the definition of the network is important. For example, Sacerdote (2001) finds that only students’ individual room-mates affect their college performance, while a broader set of peers matters for decisions related to social group participation; Renna et al. (2008) document that adolescents’ weights are more responsive to those of their friends of the same gender, while Patacchini et al. (2016) find that adolescent friendships lasting longer than 1 year have persistent effects on adolescents’ education outcomes, while shorter-lived friendships do not. Measurement error on the network will also bias parameter estimates. A more detailed overview of this issue, as well as of methods to deal with it, is provided in Advani and Malde (2014) and another companion paper (Advani and Malde 2016).

To illustrate the practical restrictions imposed by each of the different models, empirical specifications and conditions for causal identification, we will use a simple and widely studied question in the education and labour economics literatures: How is a teenager’s schooling performance influenced by his friends? This is also a question of great policy interest.^4^ More specifically, this paper will provide an overview of methods that can yield answers to questions such as ‘Is a teenager’s schooling performance influenced by the average schooling performance of her friends?’; ‘Do teenagers gain more utility from studying if their friends also study?’; ‘What is the relative importance of the average schooling performance of a teenager’s friends, and of complementarities arising from one’s friends’ studying decisions in shaping a teenager’s overall schooling performance?’; and ‘How does a student’s popularity influence his/her schooling performance?’ The same methods can also be applied to answer analogous questions about interactions between many other types of agents.

The literature on methods for networks data is broad and developing rapidly. We therefore focus our review on the issues outlined above, leaving aside a number of other interesting areas, including methods to deal with endogenous network formation and measurement error in the network, which we survey elsewhere (Advani and Malde 2016). In addition, though many of the methods reviewed here either build on or apply methods developed in spatial econometrics, it is not our objective to provide an overview of spatial econometric methods (see instead Anselin 1988). Boucher and Fortin (2015) provide a complementary review, though they do not cover methods using experimental and quasi-experimental variation.

The rest of the paper is organised as follows. The “Notation” section outlines the notation used in this paper. The “Local average models” section provides an overview of the local average model, where the average of neighbours’ outcomes is allowed to affect an individual’s outcome. In the “Local aggregate model” section, we cover the local aggregate model, where instead it is the total of neighbours’ outcomes that matters. In the “Hybrid local models” section, we consider hybrid local models, which allow both local average and local aggregate type effects. We consider models including network statistics that are not purely local, in the “Models with network characteristics” section. Finally, we show how experimental and quasi-experimental variation can be used to identify social effects in the “Experimental variation” section, before concluding.

## Notation

We begin by outlining the notation we use throughout the paper. We define a *network* or *graph*  as a set of nodes, , and edges or links, .^5^ The nodes represent individual agents, and the edges represent the links between pairs of nodes. In economic applications, nodes are usually individuals, households, firms or countries. Edges could be social ties such as friendship, kinship, or co-working, or economic ties such as purchases, loans, or trade. The number of nodes present in *g* is , and the number of edges is . We define  as the set of all possible networks on *N* nodes.

In the simplest case—the *binary network*—any (ordered) pair of nodes  is either linked, , or not linked, . If  then *j* is described as being a *neighbour* of *i*. We denote by  the *neighbourhood* of node *i*, which contains all nodes with whom *i* is linked. Nodes that are neighbours of neighbours will be referred to as *‘second-degree neighbour’*. Typically, it is convenient to assume that . Edges may be directed, so that a link from node *i* to node *j* is not the same as a link from node *j* to node *i*; in this case, the network is a *directed graph* (or *digraph*).

Network graphs, whether directed or not, can also be represented by an *adjacency matrix*, ***G***_*g*_, with typical element *G*_*ij*,*g*_. This is an *N*_*g*_×*N*_*g*_ matrix with the leading diagonal normalised to 0. When the network is binary, *G*_*ij*,*g*_=1 if , and 0 otherwise, while for weighted graphs, *G*_*ij*,*g*_=*wei*(*i*,*j*). We will use the notation ***G***_*i*,*g*_ to denote the *i*th row of the adjacency matrix ***G***_*g*_, and $\boldsymbol {G}_{i,g}^{\prime }$ to denote its *i*th column^6^. Many models defined for binary networks make use of the row-stochastic adjacency matrix or *influence matrix*, $\tilde {\boldsymbol {G}_{g}}$, whose elements are defined as $\tilde {G}_{ij,g}=\nicefrac {G_{ij,g}}{\sum _{j}G_{ij,g}}$^7^.

In what follows, we will frequently work with data from a number of network graphs. Graphs for different networks will be indexed, in a slight abuse of notation, by *g*=1,…,*M*, where *M* is the total number of networks in the data. Node-level variables will be indexed with *i*=1,…,*N*_*g*_, where *N*_*g*_ is the number of nodes in graph *g*. Node-level outcomes will be denoted by *y*_*i*,*g*_, while exogenous covariates will be denoted by the 1×*K* vector ***x***_*i*,*g*_ and common network-level variables will be collected in the 1×*Q* vector, ***z***_*g*_.

The node-level outcomes, covariates and network-level variables can be stacked for each node in a network. In this case, we will denote the stacked *N*_*g*_×1 outcome vector as ***y***_*g*_ and the *N*_*g*_×*K* matrix stacking node-level vectors of covariates for graph *g* as ***X***_*g*_. Common network-level variables for graph *g* will be gathered in the matrix ***Z***_*g*_=***ι***_*g*_***z***_*g*_ where ***ι***_*g*_ denotes an *N*_*g*_×1 vector of ones. The adjacency and influence matrices for network *g* will be denoted by ***G***_*g*_ and $\tilde {\boldsymbol {G}}_{g}$. At times we will also make use of the *N*_*g*_×*N*_*g*_ identity matrix, ***I***_*g*_, consisting of ones on the leading diagonal, and zeros elsewhere.

Finally, we introduce notation for vectors and matrices stacking together the network-level outcome vectors, covariate matrices and adjacency matrices for all networks in the data. $\boldsymbol {Y}=\left (\boldsymbol {y}_{1}^{\prime },\ldots,\boldsymbol {y}_{M}^{\prime }\right)^{\prime }$ is an $\sum _{g=1}^{M}{N_{g}}\times 1$ vector that stacks together the outcome vectors; $\boldsymbol {G}=diag\{\boldsymbol {G}_{g}\}_{g=1}^{g=M}$ denotes the $\sum _{g=1}^{M}{N_{g}}\times \sum _{g=1}^{M}{N_{g}}$ block-diagonal matrix with network-level adjacency matrices along the leading diagonal and zeros of the diagonal, and analogously $\tilde {\boldsymbol {G}}=diag\{\tilde {\boldsymbol {G}_{g}}\}_{g=1}^{g=M}$ (with similar dimensions as ***G***) for the influence matrices; and $\boldsymbol {X}=\left (\boldsymbol {X}_{1}^{\prime },\ldots,\boldsymbol {X}_{M}^{\prime }\right)^{\prime }$ and $\boldsymbol {Z}=\left (\boldsymbol {Z}_{1}^{\prime },\ldots,\boldsymbol {Z}_{M}^{\prime }\right)^{\prime }$ are respectively, $\sum _{g=1}^{M}{N_{g}}\times K$ and $\sum _{g=1}^{M}{N_{g}}\times Q$ matrices, that stack together the covariate matrices across networks. Finally, we define the vector ***ι*** as a $\sum _{g=1}^{M}{N_{g}}\times 1$ vector of ones and the matrix $\boldsymbol {L}=diag\left \{\boldsymbol {\iota }_{g}\right \}_{g=1}^{g=M}$, as an $\sum _{g=1}^{M}{N_{g}}\times M$ matrix with each column being an indicator for being in a particular network.

## Local average models

### Setup

In local average models, an agent’s outcome (or choice) is influenced by the average outcome of its neighbours^8^. Thus, an individual’s schooling effort or performance is influenced by the average schooling effort or performance of his friends. We characterise the individual as a node, *i*, in network *g*, with outcome *y*_*i*,*g*_. This outcome is modelled as being influenced by the individual’s own observed characteristics, ***x***_*i*,*g*_, scalar unobserved heterogeneity *ε*_*i*,*g*_, observed network characteristics ***z***_*g*_, an unobserved network characteristic *ν*_*g*_, and the average outcomes and characteristics of neighbours, $\sum _{j=1}^{N_{g}}\tilde {G}_{ij,g}y_{j,g}$ and ${\sum \limits _{k=1}^{K}}{\sum \limits _{j=1}^{N_{g}}}\tilde {G}_{ij,g}x_{j,k,g}$. Below, we consider identification conditions when data are available from multiple networks, though some results apply to data from a single network^9^.

Stacking together data from multiple networks yields the following empirical specification, expressed in matrix terms:

1 $$ \boldsymbol{Y}=\alpha\boldsymbol{\iota}{+\,}\beta\tilde{\boldsymbol{G}}\boldsymbol{Y}+\boldsymbol{X}\boldsymbol{\gamma}+\tilde{\boldsymbol{G}}\boldsymbol{X}\boldsymbol{\delta}+\boldsymbol{Z}\boldsymbol{\eta}+\boldsymbol{L}\boldsymbol{\nu}+\boldsymbol{\varepsilon}  $$

where ***Y***, ***ι***, $\tilde {\boldsymbol {G}}$, ***X***, ***Z***, ***L*** and ***ν*** are as defined previously. Pre-multiplying the vector ***Y*** by $\tilde {\boldsymbol {G}}$ gives a vector containing, for each individual, the average outcome of his neighbours, and similarly $\tilde {\boldsymbol {G}}\boldsymbol {X}$ is a vector of the average characteristics of his friends. The social effect of interest is *β*, the effect of an increase in the mean of neighbours’ outcome on the individual’s outcome. This is often described as the ‘endogenous social effect’, in contrast to the (vector of) ‘contextual effect(s)’ (or ‘exogenous social effect(s)’), ***δ***, which represent the effect of an increase in neighbours’ *characteristics*. ***ν*** is described as the ‘correlated effect’, capturing the correlation in individuals’ outcomes due to common (unobserved) shocks.

Given the simple empirical form of this model, it has been widely applied in the economics literature. Examples include:

- Understanding how the average schooling performance of an individual’s peers influences the individual’s own performance in a setting where students share a number of different classes (e.g. De Giorgi et al. 2010), or where students have some (but not all) common friends (e.g. Bramoullé et al. 2009).
- Understanding how non-market links between firms arising from company directors being members of multiple company boards influence firm choices on investment and executive pay (e.g. Patnam 2013).

Although this specification is widely used in the empirical literature, few studies consider or acknowledge the form of its underlying economic model, even though parameter estimates are subsequently used to evaluate alternative policies and to make policy recommendations. Indeed, parameters are typically interpreted as in the econometric model of Manski (1993), whose parameters do not map back to ‘deep’ structural (i.e. policy invariant) parameters without an economic model.

### Theoretical foundations

An economic model that leads to this specification is one where nodes have a desire to conform to the average behaviour and characteristics of their neighbours (Akerlof 1980; Jones 1984; Bernheim 1994; Patacchini and Zenou 2012). In our schooling example, conformism implies that individuals would want to exert a similar amount of effort in their school work as their friends so as to ‘fit in’. Conditional on the individual’s own characteristics which affect his school effort, if his friends exert relatively low effort in their school work, he will reduce his effort. Below we write out this model more formally and demonstrate how it leads to Eq. 1.

Conformism is commonly modelled by individual payoffs that are decreasing in the distance between own outcome and network neighbours’ average outcomes^10^. Payoffs are also allowed to vary with an individual heterogeneity parameter, $\pi _{i,g}(\boldsymbol {X}_{g},\tilde {\boldsymbol {G}}_{i,g})$, which captures the individual’s ability or productivity associated with the outcome:

2 $${} \begin{aligned} U_{i}\left(y_{i,g};\boldsymbol{y}_{-i,g},\boldsymbol{X}_{g},\tilde{\boldsymbol{G}}_{i,g}\right)&=\left(\vphantom{\sum\limits_{j=1}^{N_{g}}}\pi_{i,g}\left(\boldsymbol{X}_{g},\tilde{\boldsymbol{G}}_{i,g}\right)_{i,g}\right.\\ &-\frac{1}{2}\left(\vphantom{\sum\limits_{j=1}^{N_{g}}}y_{i,g} \left. - 2\beta\sum\limits_{j=1}^{N_{g}}\tilde{G}_{ij,g}y_{j,g}\right)\right)y_{i,g} \end{aligned}  $$

*β* in Eq. 2 can be thought of as a taste for conformism. Although we write this model as though individuals are perfectly able to observe each others’ actions, this assumption can be relaxed. In particular, an econometric specification similar to Eq. 1 can be obtained from a static model with imperfect information (see Blume et al. 2015).

The best response function derived from the first order condition with respect to *y*_*i*,*g*_ is:

3 $$ y_{i,g}=\pi_{i,g}\left(\boldsymbol{X}_{g},\tilde{\boldsymbol{G}}_{i,g}\right)+\beta\sum_{j=1}^{N_{g}}\tilde{G}_{ij,g}y_{j,g}  $$

Patacchini and Zenou (2012) derive the conditions under which a Nash equilibrium exists, and characterise properties of this equilibrium.

Note that this is not the only economic model that leads to an empirical specification of this form: a similar specification arises from, for example, models of perfect risk sharing^11^. Here, when preferences are homogeneous and risk is perfectly shared, the consumption of risk-averse households will co-move with the average household consumption in the risk sharing group or network Townsend (1994).

The individual heterogeneity parameter, $\pi _{i,g}(\boldsymbol {X}_{g},\tilde {\boldsymbol {G}}_{i,g})$, can be modelled as a linear function of observed and unobserved individual and network characteristics:

4 $$ \pi_{i,g}\left(\boldsymbol{X}_{g},\tilde{\boldsymbol{G}}_{i,g}\right)=\boldsymbol{x}{}_{i,g}\boldsymbol{\gamma}+\sum_{j=1}^{N_{g}}\tilde{G}_{ij,g}\boldsymbol{x}{}_{j,g}\boldsymbol{\delta}+\boldsymbol{z}_{g}\boldsymbol{\eta}+\nu_{g}+\varepsilon_{i,g}  $$

Substituting for this in Eq. 3, we obtain the following best response function for individual outcomes:

5 $$ y_{i,g}=\beta\sum_{j=1}^{N_{g}}\tilde{G}_{ij,g}y_{j,g}+\boldsymbol{x}{}_{i,g}\boldsymbol{\gamma}+\sum_{j=1}^{N_{g}}\tilde{G}_{ij,g}\boldsymbol{x}{}_{j,g}\boldsymbol{\delta}+\boldsymbol{z}_{g}\boldsymbol{\eta}+\nu_{g}+\varepsilon_{i,g}  $$

Then, stacking observations for all nodes in multiple networks, we obtain Eq. 1, which can be taken to the data.

### Identification

#### Without network fixed effects

Bramoullé et al. (2009) study the identification and estimation of Eq. 1 in observational data with detailed network information or data from partially overlapping peer groups.^12^ To proceed further, one needs to make some assumptions on the relationship between the unobserved variables— ***ν*** and ***ε***—and the other right-hand side variables in Eq. 1.

We first consider identification under the assumptions that $\mathbb {E}\big [\boldsymbol {\varepsilon }|\boldsymbol {X},\boldsymbol {Z},\tilde {\boldsymbol {G}}\big ]=0$ and $\mathbb {E}\big [\boldsymbol {\nu }|\:\boldsymbol {X},\boldsymbol {Z},\tilde {\boldsymbol {G}}\big ]=0$, i.e. both the individual level error term, ***ε*** and the network level unobservable are assumed to be mean independent of the observed individual and network-level characteristics and of the network. We will later relax the assumption on ***ν***.

Under these assumptions, the parameters {*α*,*β*,***γ***,***δ***,***η***} are identified if $\left \{\boldsymbol {I},\,\tilde {\boldsymbol {G}},\,\tilde {\boldsymbol {G}}^{2}\right \}$ are linearly independent. Identification thus relies on the network structure. In particular, the condition would not hold in networks composed only of cliques—subnetworks comprising of completely connected components—of the same size, and where the diagonal terms in the influence matrix, $\tilde {\boldsymbol {G}}$ are not set to 0. In this case, $\tilde {\boldsymbol {G}}^{2}$ can be expressed as a linear function of ***I*** and $\tilde {\boldsymbol {G}}$. Moreover, the model is then similar to the single peer group case of Manski (1993), and the methods outlined in Blume et al. (2010) apply.

**Fig. 1:**
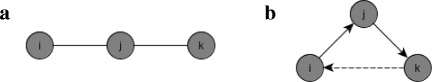
Intransitive triad in a undirected network (**a**, left panel) and a directed network (**b**, right panel)

In an undirected network (such as that in the left panel in Fig. 1 below), this identification condition holds when there exists a triple of nodes (*i*, *j*, *k*) such that *i* is connected to *j* but not *k*, and *j* is connected to *k*. The exogenous characteristics of *k*, ***x***_*k*,*g*_, directly affect *j*’s outcome, but not (directly) that of *i*, hence forming valid instruments for the outcome of *i*’s neighbours (i.e. *j*’s outcome) in the equation for node *i*. Intuitively, this method uses the characteristics of second-degree neighbours who are not direct neighbours as instruments for outcomes of direct neighbours.

It is thus immediately apparent why identification fails in networks composed only of cliques: in such networks, there is no triple of nodes (*i*, *j*, *k*) such that *i* is connected to *j*, and *j* is connected to *k*, but *i* is not connected to *k*.

In the directed network case, the condition is somewhat weaker, requiring only the presence of an intransitive triad: that is, a triple such that ,  and  (as in the right panel of Fig. 1 above).^13^ This is weaker than in undirected networks, which would also require that .

These conditions impose strong restrictions on behaviour. The identification condition for undirected networks, for example, relies on *i* and *k* not influencing one another directly, which might be too strong an assumption in contexts such as within-classroom networks, where it is not unreasonable to assume that all students are likely to interact with one another, and hence influence one another. Similarly, the identification assumption in a directed network is likely to hold in specific contexts only: for example, the effort of check-out workers who face the same direction (or are arranged in a circle) would be influenced by that of the colleagues that they can see, satisfying the identification condition (Mas and Moretti 2009).

Let us consider how this method could be applied to identify the influence of the average schooling performance of an individual’s friends on the individual. Potential variables that one might include as controls include the individual’s age, gender, and parental income; the average age, gender, and parental income of his friends; and some observed school characteristics such as expenditure per pupil. Assume first that the underlying friendship network in this school is undirected as in the left panel of Fig. 1, so that if *i* considers *j* to be his friend, *j* also considers *i* to be his friend. *j* also has a friend *k* who is not friends with *i*. We could then use the age, gender, and parental income of *k* as instruments for the schooling performance of *j* in the equation for *i*. If instead, the network were directed as in the right panel of Fig. 1, where the arrows indicate who is affected by whom (i.e. *i* is affected by *j* in the Figure, and so on), we can still use the age, gender, and parental income of *k* as instruments for the school performance of *j* in the equation for *i* even though *k* is connected with *i*. This is possible since the direction of the relationship is such that *k*’s school performance is affected by *i*’s performance, but the converse is not true.

#### Including network fixed effects

The identification result above requires that the network-level unobservable term be mean independent of the observed covariates, ***X*** and ***Z***, and of the network, $\tilde {\boldsymbol {G}}$. However, in many circumstances, one might be concerned that unobservable characteristics of the network might be correlated with ***X***, so that $\mathbb {E}\big [\boldsymbol {\nu }|\boldsymbol {X},\boldsymbol {Z},\tilde {\boldsymbol {G}}\big ]\neq 0$. In the schooling context, where the network of interest is often constrained to be within the same school, a large literature (e.g. Black 1999; Gibbons and Machin 2003; Bayer et al. 2007) indicates that wealthier parents choose to live in areas with good schools, making it likely that children with higher parental income will be in schools with teachers who have better unobserved teaching abilities. A natural solution is to include network fixed effects, $\boldsymbol {L}\tilde {\boldsymbol {\nu }}$, which will control for the network-level unobservable, ***Lν***, though at the cost of allowing us to identify ***η***.

Since the fixed effects themselves are generally not of interest, to ease estimation they are removed using a *within transformation*. This is done by pre-multiplying Eq. 1 by ***J***, a block diagonal matrix that stacks the network-level transformation matrices $\boldsymbol {J}_{g}=\boldsymbol {I}_{g}-\frac {1}{N_{g}}\big (\boldsymbol {\iota }_{g}\boldsymbol {\iota }_{g}^{\prime }\big)$ along the leading diagonal, and off-diagonal terms are set to zero. This subtracts the network-level mean of the outcome, giving a vector ***JY*** of deviations from the mean. The resulting model is of the following form:

6 $$ \boldsymbol{J}\boldsymbol{Y}=\beta\boldsymbol{J}\tilde{\boldsymbol{G}}\boldsymbol{Y}+\boldsymbol{J}\boldsymbol{X}\boldsymbol{\gamma}+\boldsymbol{J}\tilde{\boldsymbol{G}}\boldsymbol{X}\boldsymbol{\delta}+\boldsymbol{J}\boldsymbol{\varepsilon}  $$

**Fig. 2:**
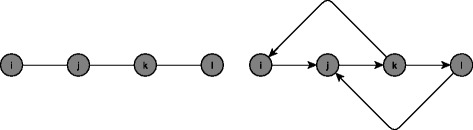
Identification with network fixed effects. The picture on the left panel shows an undirected network with an agent *l* who is at least three steps away from *i*, while the picture on the right panel shows the same for a directed network

The identification condition here imposes a stronger requirement on network structure: now, the matrices $\left \{\boldsymbol {I},\,\tilde {\boldsymbol {G}},\,\tilde {\boldsymbol {G}}^{2},\,\tilde {\boldsymbol {G}}^{3}\right \}$ need to be linearly independent. This requires that there exists a pair of agents (*i*, *l*) such that the shortest path between them is of length 3. That is, *i* would need to go through at least two other nodes to get to *l* (as in Fig. 2 below). The presence of at least two intermediate agents allows the researcher to use the characteristics of third-degree neighbours (neighbours-of-neighbours-of-neighbours who are not direct neighbours or neighbours-of-neighbours) as an additional instrument to account for the network fixed effect.

A concern that arises when applying this method is that of instrument strength. Bramoullé et al. (2009) find that this varies with graph *density*, i.e. the proportion of node pairs that are linked; and the level of *clustering*, i.e. the proportion of node triples such that precisely two of the possible three edges are connected.^14^ Instrument strength is declining in density, since the number of intransitive triads tends to zero, reducing the variation in the instrument. The results for clustering are non-monotone and depend on density.

The discussion thus far has assumed that the network through which the endogenous social effect operates is the same as the network through which the contextual effect operates. It is possible to allow for these two networks to be distinct. This could be useful in the school setting, for instance, where contextual effects could be driven by the average characteristics of all students in the school, while endogenous effects by the outcomes of a subset of students who are friends. Such a formulation allows for a more flexible representation of the environment: the contextual effect could operate through, for example, the resources available to a school, which might depend on the parental income of all students (if schools are financed through local taxation), while peer influences on effort or performance might come only from friends.

Let ***G***_***X***,*g*_ and ***G***_***y***,*g*_ denote the network-level adjacency matrices through which, respectively, the contextual and endogenous effects operate. As before, we define the block diagonal matrices $\boldsymbol {G}_{\boldsymbol {X}}=diag \left \{\boldsymbol {G}_{\boldsymbol {X},g}\right \}_{g=1}^{g=M}$ and $\boldsymbol {G}_{\boldsymbol {y}}=diag \left \{\boldsymbol {G}_{\boldsymbol {y},g}\right \}_{g=1}^{g=M}$. Blume et al. (2015) study identification of this model assuming that both networks are (conditionally) exogenous and show that when the matrices ***G***_***y***_ and ***G***_***X***_ are observed by the econometrician, and at least one of *δ* and *γ* is non-zero, then the necessary and sufficient conditions for the parameters of Eq. 1 to be identified are that the matrices ***I***, ***G***_***y***_, ***G***_***X***_ and ***G***_***y***_***G***_***X***_ are linearly independent.

## Local aggregate model

The local aggregate class of models, studied theoretically by Ballester et al. (2006), Calvó-Armengol et al. (2009), and Bramoullé et al. (2014), and empirically by Calvó-Armengol et al. (2009), Lee and Liu (2010), and Liu et al. (2012), considers settings where agents’ utilities are a function of the aggregate outcomes (or choices) of their neighbours. This is in contrast with local average models, where utilities are a function of the difference between own outcome and the average outcome of one’s neighbours. The local aggregate model thus encompasses a different assumption on behaviour—it applies to situations where there are strategic complementarities or strategic substitutabilities—and allows dyad-level social effects to aggregate at a network or group level.

Examples of cases where strategic complementarities and substitutabilities are likely to be at play and thus where a local aggregate, rather than local average model, would be more appropriate include:

- An individual’s costs of engaging in crime may be lower when his neighbours also engage in crime (e.g. Bramoullé et al. 2014).^15^
- An agent is more likely to learn about a new product and how it works if more of his neighbours know about it and have used it.
- A student gains more utility from undertaking effort in studying if his friends also undertake more effort (e.g. Calvó-Armengol et al. 2009).

### Setup and theoretical foundations

The local aggregate model corresponds to the following empirical specification:

7 $$ \boldsymbol{Y}=\alpha\boldsymbol{\iota}{+\,}\beta\boldsymbol{G}\boldsymbol{Y}+\boldsymbol{X}{\boldsymbol{\gamma}}+\boldsymbol{\tilde{G}}\boldsymbol{X}{\boldsymbol{\delta}}+\boldsymbol{Z}\boldsymbol{\eta}+\boldsymbol{L}\boldsymbol{\nu}+{\boldsymbol{\varepsilon}}  $$

where ***Y***, ***G***, ***X*** and ***Z*** are as defined earlier. In contrast to the local average model, $\tilde {\boldsymbol {G}}$ has been replaced by ***G***. As before, the social effect of interest is *β*, which now represents the effect of the aggregate outcome of one’s neighbours on one’s own outcome.

This specification can be motivated by the best responses of a game in which nodes have linear-quadratic utility and there are strategic complementarities or substitutabilities between the actions of a node and those of its neighbours. A model of this type has been studied by among others, Ballester et al. (2006) and Bramoullé et al. (2014). The former focus on the case of strategic complementarities, while the latter study the case with strategic substitutabilities and characterise all equilibria of this game. In this model, the utility function for agent *i* in network *g* takes the following form:

8 $$ \begin{aligned} {}U_{i}\left(y_{i,g};\boldsymbol{y}_{-i,g},\boldsymbol{X}_{g},\boldsymbol{G}_{g}\right)&=\left({\vphantom{\sum\limits_{j=1}^{N_{g}}}}\pi_{i,g}\left(\boldsymbol{X}_{g},\tilde{\boldsymbol{G}}_{i,g}\right)\right.\\ &-\frac{1}{2}\left(\vphantom{\sum\limits_{j=1}^{N_{g}}}y_{i,g} \left.-2\beta\sum\limits_{j=1}^{N_{g}}G_{ij,g}y_{j,g}\right)\right)y_{i,g} \end{aligned}  $$

where *y*_*i*,*g*_ is *i*’s action or choice, and $\pi _{i,g}(\boldsymbol {X}_{g},\tilde {\boldsymbol {G}}_{i,g})$ is, as before, an individual heterogeneity parameter^16^. This has the same form as Eq. 2, but with ***G*** replacing $\tilde {\boldsymbol {G}}$. $\pi _{i,g}(\boldsymbol {X}_{g},\tilde {\boldsymbol {G}}_{i,g})$ is again typically parameterised as:

$$\pi_{i,g}\left(\boldsymbol{X}_{g},\tilde{\boldsymbol{G}}_{i,g}\right)=\boldsymbol{x}_{i,g}\boldsymbol{\delta}+\sum_{j=1}^{n}\tilde{G}_{ij,g}\boldsymbol{x}_{j,g}\boldsymbol{\gamma}+\boldsymbol{z}_{g}\boldsymbol{\eta}+\nu_{g}+\varepsilon_{i,g} $$

so that individual heterogeneity is a function of a node’s own characteristics, the *average* characteristics of its neighbours, network-level observed characteristics, and some unobserved network- and individual-level terms. This model shares some features with the model of Blume et al. (2015), as different network matrices are used to capture the effects of neighbours’ outcomes and characteristics, which helps to ease identification.

The quadratic cost of own actions means that in the absence of any network, there would be a unique optimal amount of effort the node would exert, as in the local average model. *β*>0 implies that neighbours’ actions are complementary to a node’s own actions, so that the node increases his actions in response to those of his neighbours. If *β*<0, then nodes’ actions are substitutes, and the reverse is true. Nodes choose *y*_*i*,*g*_ so as to maximise their utility.

The best response function is:

9 $${} {\begin{aligned} y_{i,g}^{*}\left(\boldsymbol{G}_{g}\right)=\beta\sum_{j=1}^{n}G_{ij,g}y_{j,g}+\boldsymbol{x}_{i,g}\boldsymbol{\delta}+\sum_{j=1}^{n}\tilde{G}_{ij,g}\boldsymbol{x}_{j,g}\boldsymbol{\gamma}+\boldsymbol{z}_{g}\boldsymbol{\eta}+\nu_{g}+\varepsilon_{i,g} \end{aligned}}  $$

Ballester et al. (2006) solve for the Nash equilibrium of this game when *β*>0 and show that when | *βω*_*max*_(***G***_*g*_)| <1, where *ω*_*max*_(***G***_*g*_) is the largest eigenvalue of the matrix ***G***_*g*_, the equilibrium is unique and the equilibrium outcome relates to a node’s Katz-Bonacich centrality, which is defined as ***b***(***G***_*g*_,*β*)=(***I***_*g*_−*β****G***_*g*_)^−1^(***ι***_*g*_)^17^^,^^18^.

Bramoullé et al. (2014) study the game with strategic substitutabilities between the action of a node and those of its neighbours. In this case, when one agent chooses one action, his neighbours would choose the opposite action, inducing their neighbours to choose a similar action to the first agent, and so on. In equilibrium, the agent’s choice is influenced by the direct choice of his neighbours, as well as by the aggregated sum of these opposing choices across different network paths. Bramoullé et al. (2014) characterise the set of Nash equilibria of the game and show that they depend on the lowest eigenvalue of the network adjacency matrix, *ω*_*min*_(***G***_*g*_). This eigenvalue is negative and relates to the aggregated effect of agents’ choices on others. When it is large in magnitude, the opposing forces outlined above could go in many different directions, leading to multiple equilibria. When it is sufficiently small, i.e. *β*|*ω*_*min*_(***G***_*g*_)|<1, a unique equilibrium exists since the opposing forces ‘balance out’ to converge to a single point. When multiple equilibria are possible, they must be accounted for in any empirical analysis. Methods developed in the literature on the econometrics of games may be applied here (Bisin et al. 2011). See de Paula (2013) for an overview. Below, we focus on conditions under which the social effect parameter is identified when a unique equilibrium exists.

When a unique equilibrium exists, this theoretical setup implies the following empirical model (stacking data from multiple networks):

10 $$ \boldsymbol{Y}=\alpha\boldsymbol{\iota}+\beta\boldsymbol{G}\boldsymbol{Y}+\boldsymbol{X}\boldsymbol{\gamma}+\tilde{\boldsymbol{G}}\boldsymbol{X}\boldsymbol{\delta}+\boldsymbol{Z}\boldsymbol{\eta}+\boldsymbol{L}\boldsymbol{\nu}+\boldsymbol{\varepsilon}  $$

where variables and parameters are as defined above.

### Identification

Identification of Eq. 10 using observational data has been studied by Calvó-Armengol et al. (2009), Lee and Liu (2010) and Liu et al. (2012). They proceed under the assumption that $\mathbb {E}\big [\boldsymbol {\varepsilon }|\boldsymbol {X},\boldsymbol {Z},\boldsymbol {G},\tilde {\boldsymbol {G}}\big ]\,=\,0$ and $\mathbb {E}\big [\boldsymbol {\nu }|\boldsymbol {X},\boldsymbol {Z},\boldsymbol {G},\tilde {\boldsymbol {G}}\big ]\!\neq \!0$. That is, the node-varying error component is conditionally mean independent of node- and network-level observables and of the network, while the network-level unobservable could be correlated with node- and network-level characteristics and/or the network itself.

These assumptions imply a two-stage network formation process. First, agents select into a network based on a set of observed individual- and network-level characteristics and some common network-level unobservables. Then in a second stage, they form links with other nodes. There are no network-level unobservable factors that determine link formation once the network has been selected by the node. Moreover, there are no node-level unobservable factors that determine the choice of network or link formation within the chosen network.

To proceed, we assume that data are available for multiple networks. Then, as in the “Local average models” section, we replace the network-level observables, ***Z***, and the network-level unobservable, ***Lν*** in Eq. 10 with network fixed effects, $\boldsymbol {L}\tilde {\boldsymbol {\nu }}$, where $\tilde {\boldsymbol {\nu }}$ is a *M*×1 vector that captures the network fixed effects.

To account for the fixed effect, a within-transformation is applied, as in the “Local average models” section. This transformation is represented by the block diagonal matrix ***J*** that stacks the following network-level transformation matrices—$\boldsymbol {J}_{g}=\boldsymbol {I}_{g}-\frac {1}{N_{g}}\big (\boldsymbol {\iota }_{g}\boldsymbol {\iota }_{g}^{\prime }\big)$—along the leading diagonal, with off-diagonal terms set to 0. The resulting model, analogous to Eq. 6, is:

11 $$ \boldsymbol{J}\boldsymbol{Y}=\beta\boldsymbol{J}\boldsymbol{G}\boldsymbol{Y}+\boldsymbol{J}\boldsymbol{X}\boldsymbol{\gamma}+\boldsymbol{J}\tilde{\boldsymbol{G}}\boldsymbol{X}\boldsymbol{\delta}+\boldsymbol{J}\boldsymbol{\varepsilon}  $$

The model above suffers from the reflection problem, since ***Y*** appears on both sides of the equation. However, the parameters of Eq. 11 can be identified using linear instrumental variables (IV) if the deterministic part of the right-hand side, $[\mathbb {E}(\boldsymbol {J}\boldsymbol {G}\boldsymbol {Y}),\boldsymbol {J}\boldsymbol {X},\boldsymbol {J}\tilde {\boldsymbol {G}}\boldsymbol {X}]$, has full column rank. To see the conditions under which this is satisfied, we examine the term with the endogenous variable, $\mathbb {E}(\boldsymbol {J}\boldsymbol {G}\boldsymbol {Y})$. Under the assumption that |*βω*_*max*_(***G***_*g*_)|<1, we obtain the following from the reduced form equation of Eq. 10:

12 $${} {\begin{aligned} \mathbb{E}(\boldsymbol{J}\boldsymbol{G}\boldsymbol{Y})&=\boldsymbol{J}\left(\boldsymbol{G}\boldsymbol{X}+\beta\boldsymbol{G}^{2}\boldsymbol{X}+\ldots\right)\boldsymbol{\gamma}+\boldsymbol{J}\left(\boldsymbol{G}\tilde{\boldsymbol{G}}\boldsymbol{X}+\beta\boldsymbol{G}^{2}\tilde{\boldsymbol{G}}\boldsymbol{X}+\ldots\right)\boldsymbol{\delta} \\ &+\boldsymbol{J}\left(\boldsymbol{G}\boldsymbol{L}+\beta\boldsymbol{G}^{2}\boldsymbol{L}+\ldots\right)\tilde{\boldsymbol{\nu}} \end{aligned}}  $$

We can thus see that if the matrices $\{\boldsymbol {I},{\boldsymbol {G}},{\tilde {\boldsymbol {G}}},{\boldsymbol {G}}{\tilde {\boldsymbol {G}}}\}$ are linearly independent, and ***γ***, ***δ***, and $\tilde {\boldsymbol {\nu }}$ each have some non-zero terms, the parameters of Eq. 10 are identified^19^. Node degree (***GL***), along with the sum of the exogenous characteristics of the node’s direct neighbours (***GX***), and sum of the average exogenous characteristics of its second-degree neighbours ($\boldsymbol {G}\tilde {\boldsymbol {G}}\boldsymbol {X}$) can be used as instruments for the total outcome of the node’s neighbours (***GY***). That node degree can be used as an instrument follows intuitively from the theoretical model: when there are dyad-level strategic complementarities, an individual’s own outcome will be a weakly increasing function of the number of his direct neighbours. Moreover, the availability of node degree as an instrument can allow one to identify parameters without using the exogenous characteristics, ***X***, of second- or higher-degree network neighbours, which can be particularly advantageous when only sampled data are available, as shown by Liu (2013).

In terms of practical application, consider using this method to identify whether there are complementarities between the schooling performance of an individual and that of his friends, conditional on his own characteristics (age, gender, and parental income), the composition of his friends (average age, gender, and parental income), and some school characteristics. Then, if there are individuals in the same network with different numbers of friends, and the matrices $\big \{\boldsymbol {I},{\boldsymbol {G}},{\tilde {\boldsymbol {G}}},{\boldsymbol {G}}{\tilde {\boldsymbol {G}}}\big \}$ are linearly independent, the individual’s degree, along with the total characteristics of his friends (i.e. total age, gender, and parental income) and the sum of the average age, gender, and parental income of the individual’s friends of friends can be used as instruments for the sum of the individual’s friends’ schooling performance. Similar to the local average model, this strategy relies on a researcher being able to define reasonably well direct, as well as indirect, neighbours, which might be less clear in some contexts.

Parameters can still be identified if there is no variation in node degree within a network for all networks in the data, but there is variation in degree across networks. In this case, $\boldsymbol {G}_{g}=\bar {d}_{g}\tilde {\boldsymbol {G}}_{g}$ and $\big [E(\boldsymbol {J}\boldsymbol {G}\boldsymbol {Y}),\boldsymbol {J}\boldsymbol {X},\boldsymbol {J}\tilde {\boldsymbol {G}}\boldsymbol {X}\big ]$ has full column rank if the matrices $\big \{\boldsymbol {I},\boldsymbol {G},\tilde {\boldsymbol {G}},\boldsymbol {G}\tilde {\boldsymbol {G}},\tilde {\boldsymbol {G}^{2}},\boldsymbol {G}\tilde {\boldsymbol {G}^{2}}\big \}$ are linearly independent and ***γ*** and ***δ*** each have non-zero terms^20^. This requires the presence of some pair of nodes *i* and *k*, who are only indirectly connected. Finally, when there is no variation in node degree within and across all networks in the data, parameters can be identified using a similar condition as encountered in the “Local average models” section above: the matrices $\big \{\boldsymbol {I},\,\tilde {\boldsymbol {G}},\,\tilde {\boldsymbol {G}}^{2},\,\tilde {\boldsymbol {G}}^{3}\big \}$ should be linearly independent.

**Fig. 3:**
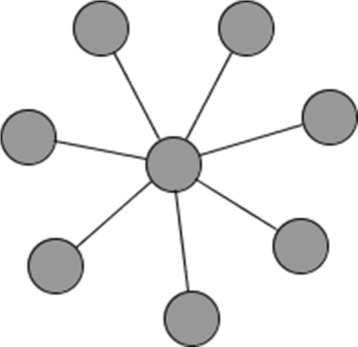
Star network

It is possible to identify model parameters in the local aggregate model in networks where the local average model parameters cannot be identified. For example, in a star network (see Fig. 3), there is no pair of agents that has a geodesic distance (i.e. shortest path) of three or more, so this fails the identification condition for the local average model with fixed effects. However, there is variation in node degree within the network and the matrices $\boldsymbol {I}_{g},{\boldsymbol {G}}_{g},{\tilde {\boldsymbol {G}}}_{g},{\boldsymbol {G}}_{g}{\tilde {\boldsymbol {G}}}_{g}$ can be shown to be linearly independent, thus satisfying the identification conditions for the local aggregate model.

## Hybrid local models

The local average and local aggregate models embody distinct mechanisms through which social effects arise. One may be interested in jointly testing these mechanisms, and empirically identifying the most relevant one for a particular context. Liu et al. (2014a) present a framework nesting both the local aggregate and local average models, allowing for this.

### Setup and theoretical foundations

The utility function for node *i* in network *g* that nests both the (linear) local aggregate and local average models has the following form:

13 $${} {\begin{aligned} U_{i}\left(y_{i,g};\boldsymbol{y}_{-i,g},\boldsymbol{X}_{g},\tilde{\boldsymbol{G}}_{i,g},\boldsymbol{G}_{i,g}\right)&=\left(\pi_{i,g}\left(\boldsymbol{X}_{g},\tilde{\boldsymbol{G}}_{i,g}\right)+\beta_{1}\sum_{j=1}^{N_{g}}G_{ij,g}y_{j,g}\right.\\ \quad &\left.-\frac{1}{2}\left(\! y_{i,g}\,-\,2\beta_{2}\sum_{j=1}^{N_{g}}\tilde{G}_{ij,g}y_{j,g}\!\right)\!\right)y_{i,g} \end{aligned}}  $$

where $\pi _{i,g}(\boldsymbol {X}_{g},\tilde {\boldsymbol {G}}_{i,g})$ is node-specific observed heterogeneity, which affects the node’s marginal return from the chosen outcome level *y*_*i*,*g*_. A node’s utility is thus affected by the choices of its neighbours through changing the marginal returns of its own choice (e.g. in a schooling context, an individual’s studying effort is more productive if his friends also study), as in the local aggregate model, and by a cost of deviating from the average choice of its neighbours (i.e. individuals face a utility cost if they study when their friends do not study), as in the local average model.

The best reply function for a node *i* nests both the local average and local aggregate terms. Liu et al. (2014a) prove that under the condition that *β*_1_≥0, *β*_2_≥0 and $d_{g}^{max}\beta _{1}+\beta _{2}<1$, where $d_{g}^{max}$ is the largest degree in network *g*, the simultaneous move game has a unique interior Nash equilibrium in pure strategies. Note that this rules out the possibility of individuals’ actions being strategic substitutes (i.e. *β*<0), as for example if one student in a group needs to supply effort on homework so that the others can copy him.

The econometric model, assuming that the node-specific observed heterogeneity parameter takes the form $\pi _{i,g}\left (\boldsymbol {X}_{g},\tilde {\boldsymbol {G}}_{i,g}\right)=\boldsymbol {x}_{i,g}\boldsymbol {\gamma }+\sum _{j=1}^{N_{g}}\tilde {G}_{ij,g}\boldsymbol {x}_{j,g}\boldsymbol {\delta }+\boldsymbol {z}_{g}\eta _{g}+\nu _{g}+\varepsilon _{i,g}$, is as follows:

14 $$ \boldsymbol{Y}=\alpha\boldsymbol{\iota}+\beta_{1}\boldsymbol{G}\boldsymbol{Y}+\beta_{2}\tilde{\boldsymbol{G}}\boldsymbol{Y}+\boldsymbol{X}\boldsymbol{\gamma}+\tilde{\boldsymbol{G}}\boldsymbol{X}\boldsymbol{\delta}+\boldsymbol{Z}\boldsymbol{\eta}+\boldsymbol{L}\boldsymbol{\nu}+\boldsymbol{\varepsilon}  $$

using the same notation as before.

With data from only a single network it is not possible to separately identify *β*_1_ and *β*_2_ and hence test between the local aggregate and local average models (or indeed find that the truth is a hybrid of the two effects). Identification of parameters is considered when data from multiple networks are available under the assumption that $\mathbb {E}\big [\varepsilon _{i,g}|\boldsymbol {X}_{g},\boldsymbol {Z}_{g},\boldsymbol {G}_{g},\tilde {\boldsymbol {G}_{g}}\big ]=0$ and $\mathbb {E}\big [\boldsymbol {\nu }_{g}|\boldsymbol {X}_{g},\boldsymbol {Z}_{g},\boldsymbol {G}_{g},\tilde {\boldsymbol {G}}_{g}\big ]\neq 0$. Thus, as in the “Local average models” and “Local aggregate model” sections above, the individual error term, *ε*_*i*,*g*_ is assumed to be mean independent of node- and network-level observable characteristics and the network. The network-level unobservable, ***ν***_*g*_, by contrast is allowed to be correlated with node- and network-level characteristics and/or the network.

### Identification

To proceed, as in the local average and local aggregate model, ***Zη*** and ***Lν*** are replaced by a network-level fixed effect, $\boldsymbol {L}\tilde {\boldsymbol {\nu }}$, which is then removed using the within-transformation, ***J***. The resulting transformed network model is:

15 $$ \boldsymbol{J}\boldsymbol{Y}=\beta_{1}\boldsymbol{J}\boldsymbol{G}\boldsymbol{Y}+\beta_{2}\boldsymbol{J}\tilde{\boldsymbol{G}}\boldsymbol{Y}+\boldsymbol{J}\boldsymbol{X}\boldsymbol{\gamma}+\boldsymbol{J}\tilde{\boldsymbol{G}}\boldsymbol{X}\boldsymbol{\delta}+\boldsymbol{J}\boldsymbol{\varepsilon}  $$

When there is variation in the degree within a network *g*, the reduced form equation of Eq. 15 implies that $\boldsymbol {J}\boldsymbol {G}(\boldsymbol {I}-\beta _{1}\boldsymbol {G}-\beta _{2}\tilde {\boldsymbol {G}})^{-1}\boldsymbol {L}$ can be used as an instrument for the local aggregate term ***JGY*** and $\boldsymbol {J}\tilde {\boldsymbol {G}}(\boldsymbol {I}-\beta _{1}\boldsymbol {G}-\beta _{2}\tilde {\boldsymbol {G}})^{-1}\boldsymbol {L}$ can be used as an instrument for the local average term $\boldsymbol {J}\tilde {\boldsymbol {G}}\boldsymbol {Y}$. The model parameters may in principle thus be identified even if there are no node-level exogenous characteristics, ***X***, in the model, as long as *β*_1_≠0. However, if *β*_1_=0, the model excluding exogenous characteristics, ***X***, is tautological—in this case one is simply regressing individuals’ outcomes on the mean of the outcomes (see Angrist 2014, for further discussion). The availability of such characteristics offers more possible IVs: in particular, the total and average exogenous characteristics of direct and indirect neighbours can be used as instruments. These are necessary for identification when all nodes within a network have the same degree, though average degree may vary across networks. In this case, parameters can be identified if the matrices $\big \{\boldsymbol {I},{\boldsymbol {G}},{\tilde {\boldsymbol {G}}},{\boldsymbol {G}}{\tilde {\boldsymbol {G}}},\tilde {\boldsymbol {G}^{2}},\boldsymbol {G}\tilde {\boldsymbol {G}^{2}},\tilde {\boldsymbol {G}^{3}}\big \}$ are linearly independent^21^. If, however, all nodes in all networks have the same degree, it is not possible to identify separately the parameters *β*_1_ and *β*_2_.

This specification nests both the local average and local aggregate models, so a J-test for non-nested regression models can be applied to uncover the relevance of each mechanism. The intuition underlying the J-test is as follows: if a model is correctly specified (in terms of the set of regressors), then the fitted value of an alternative model should have no additional explanatory power in the original model, i.e. its coefficient should not be significantly different from zero. Thus, to identify which of the local average or local aggregate mechanisms is more relevant for a specific outcome, one could first estimate one of the models (e.g. the local average model), and obtain the predicted outcome value under this mechanism. In a second step, estimate the other model (in our example, the local aggregate model) and include as a regressor the predicted value from the other (i.e. local average) model. If the mechanism underlying the local average model is also relevant for the outcome, the coefficient on the predicted value will be statistically different from 0. The converse can also be done to test the relevance of the second model (the local aggregate model in our case) (see Liu et al. (2014a) for more details).

## Models with network characteristics

The models considered thus far allow for a node’s outcomes to be influenced only by outcomes of its neighbours, so-called local models. However, the broader network structure may affect node- and aggregate network- outcomes through more general functionals or features of the network. Depending on the theoretical model used, there are different predictions on which network features relate to different outcomes of interest. For example, the DeGroot (1974) model of social learning implies that a node’s eigenvector centrality, which measures its ‘importance’ in the network by how important its neighbours are, determines how influential it is in affecting the beliefs of other nodes.

Empirical testing and verification of the predictions of these theoretical models has greatly lagged the theoretical literature due to a lack of datasets with both information on network structure and socio-economic outcomes of interest. The recent availability of detailed network data from many contexts has begun to relax this constraint.

The following types of specification are typically estimated when assessing how outcomes vary with network structure, for node-level outcomes:

16 $$ \boldsymbol{Y}=\boldsymbol{f}_{\boldsymbol{y}}(\boldsymbol{w}_{\boldsymbol{y}}(\boldsymbol{G},\,\boldsymbol{Y}),\boldsymbol{X},\boldsymbol{w}_{\boldsymbol{x}}(\boldsymbol{G},\,\boldsymbol{X}),\boldsymbol{Z})+\boldsymbol{\varepsilon}  $$

and network-level outcomes:

17 $$ \bar{\boldsymbol{Y}}=\boldsymbol{f}_{\bar{\boldsymbol{y}}}(\bar{\boldsymbol{w}}_{\bar{\boldsymbol{y}}}(\boldsymbol{G}),\bar{\boldsymbol{X}},\bar{\boldsymbol{w}}_{\bar{\boldsymbol{x}}}(\boldsymbol{G},\,\bar{\boldsymbol{X}}))+\boldsymbol{u}  $$

***f***_***y***_(.) and $\boldsymbol {f}_{\bar {\boldsymbol {y}}}(.)$ are functions that specify the shape of the relationship between the network statistics and the node- and network-level outcomes. Though, in principle, the shape of ***f***_***y***_(.) should be guided by theory (where possible), through the rest of this section, we take ***f***_***y***_(.) to be a linear index in its argument, as is common in the literature. ***w***_***y***_(***G***, ***Y***) is an $\left (\sum _{g=1}^{M}{N_{g}}\times R\right)$ matrix stacking the (1×*R*) node-level vector of network statistics that vary at the node or network level and that may be interacted with ***Y***^22^. $\bar {\boldsymbol {w}}_{\bar {\boldsymbol {y}}}(\boldsymbol {G})$ is an $(M\times \bar {R})$ matrix containing the $\bar {R}$ network statistics in the network-level regression. ***X*** is a matrix of observable characteristics of nodes, ***w***_***x***_(***G***, ***X***) interacts network statistics with exogenous characteristics of nodes, and ***Z*** and $\bar {\boldsymbol {X}}$ are network-level observable characteristics. $\bar {\boldsymbol {w}}_{\bar {\boldsymbol {x}}}(\boldsymbol {G},\,\bar {\boldsymbol {X}})$ interacts network statistics with network-level observable characteristics.

The complexity of networks poses an important challenge in understanding how outcomes vary with network structure. In particular, there are no sufficient statistics that fully describe the structure of a network. For example, networks with the same average degree may vary greatly on dimensions such as density, clustering and average path length among others. Moreover, the adjacency matrix, ***G***, which describes fully the structure of a network, is too high-dimensional an object to include directly in tests of the influence of broader features of network structure. Theory can provide guidance on which statistics are likely to be relevant, and also on the shape of the relationship between the network statistic and the outcome of interest. A limitation though is that theoretical results may not be available (given currently known techniques) for outcomes one is interested in studying. This is a challenge faced by, for instance Alatas et al. (2016) who study how network structure affects information aggregation.

Below we outline methods that have been applied to analyse the effects of features of network structure on socio-economic outcomes. We do so separately for node-level specifications and network-level specifications. This literature is very much in its infancy and few methods have been developed to allow for identification of causal parameters.

### Node-level specifications

Many theoretical models predict how node-level outcomes vary with the ‘position’ of a node in the network, captured by node varying network statistics such as centrality; or with features of the node’s local neighbourhood such as node clustering; or with the ‘connectivity’ of the network, represented by statistics that vary at the network level such as network density.

A common type of empirical specification used in the literature correlates network statistics with some relevant socio-economic outcome of interest. This approach is taken by, for example, Jackson et al. (2012) who test whether informal favours take place across edges that are supported (i.e. that nodes exchanging a favour have a common neighbour), which is the prediction of their theoretical model.

This corresponds with ***w***_***y***_(***G***,***Y***) in Eq. 16 above being defined as ***w***_***y***_(***G***,***Y***)=***ω***, where ***ω*** is the matrix of network statistics of interest, and ***w***_***x***_(***G***,***X***) being defined as ***ι***. Here, ***w***_***y***_(***G***,***Y***) is defined to be a function of the network only.

When ***f***_***y***_(.) is linear, the specification is as follows:

18 $$ \boldsymbol{Y}=\alpha\boldsymbol{\iota}\mathbf{+}\boldsymbol{\omega}\boldsymbol{\beta}+\boldsymbol{X}{\boldsymbol{\gamma}}+{\boldsymbol{Z}}\boldsymbol{\eta}+{\boldsymbol{\varepsilon}}  $$

where the variables and parameters are as defined above and the parameter of interest is ***β***. Defining ***W***=(***ω***,***X***,***Z***), the key identification assumption is that E[***ε***^′^***W***]=0, that is that the right-hand side terms are uncorrelated with the error term. This may not be satisfied if there are unobserved factors that affect both the network statistic (through affecting network formation decisions) and the outcome, ***Y*** or if the network statistic is mismeasured. Both of these are important concerns that we cover in detail in Advani and Malde (2016).

In some cases, one may also be interested in estimating a model where an agent’s outcome is affected by the outcomes of his neighbours, weighted by a measure of their network position. For example, in the context of learning about a new product or technology, the DeGroot (1974) model of social learning implies that nodes’ eigenvector centrality determines how influential they are in influencing others’ behaviour. Thus, conditional on the node’s eigenvector centrality, its choices may be influenced more by the choices of his neighbours with high eigenvector centrality. Thus, one may want to weight the influence of neighbours’ outcomes on own outcomes by their eigenvector centrality, conditional on own eigenvector centrality. If we model this linearly, it implies a model of the following form:

19 $$ \boldsymbol{Y}=\alpha\boldsymbol{\iota}\mathbf{+}\boldsymbol{w}_{\boldsymbol{y}}(\boldsymbol{G},\,\boldsymbol{Y})\boldsymbol{\beta}+\tilde{\boldsymbol{X}}{\tilde{\boldsymbol{\gamma}}}+\boldsymbol{w}_{\boldsymbol{x}}(\boldsymbol{G},\,\tilde{\boldsymbol{X}})\tilde{{\boldsymbol{\delta}}}+{\boldsymbol{Z}}\boldsymbol{\eta}+\boldsymbol{L}\boldsymbol{\nu}+{\boldsymbol{\varepsilon}}  $$

***w***_***y***_(***G***, ***Y***) is an $\sum _{g}N_{g}\times R$ matrix, with the (*i*,*r*)^*th*^ element being the weighted sum of *i*’s neighbours’ outcomes, ${\sum _{j\neq i}}G_{ij,g}y_{j,g}\omega _{j,g}^{r}$ or ${\sum _{j\neq i}}\tilde {G}_{ij,g}y_{j,g}\omega _{j,g}^{r}$, with weights $\omega _{j,g}^{r}$ being the neighbour’s *r*^*th*^ network statistic (e.g. the neighbour’s eigenvector centrality in the DeGroot model of social learning). $\tilde {\boldsymbol {X}}=\left (\tilde {\boldsymbol {X}}_{1}^{\prime },\tilde {\boldsymbol {X}}_{2}^{\prime },\ldots,\tilde {\boldsymbol {X}}_{M}^{\prime }\right)^{\prime }$, where $\tilde {\boldsymbol {X}}_{g}=(\boldsymbol {X}_{g},\boldsymbol {\omega }_{g})$ is a matrix stacking together the network-level matrices of exogenous explanatory variables and (own) network statistics of interest. $\boldsymbol {w}_{\boldsymbol {x}}(\boldsymbol {G},\,\tilde {\boldsymbol {X}})$ could be defined as $\boldsymbol {G}\tilde {\boldsymbol {X}}$ or $\tilde {\boldsymbol {G}}\tilde {\boldsymbol {X}}$. Note that this formulation allows for the influence of neighbours’ background characteristics on the outcome to be weighted by the values of their network statistics. Identification of parameters in this case is complicated by the fact that ***w***_***y***_(***G***, ***Y***) is a (possibly non-linear) function of ***Y***, and thus endogenous. It may be possible to achieve identification using network-based instrumental variables, as above, though it is not immediately obvious how such an IV could be constructed. Further research is needed to shed light on these issues.

### Network-level specifications

Aggregate network-level outcomes, such as the degree of risk sharing or the aggregate penetration of a new product, may also be affected by how ‘connected’ the network is, or the ‘position’ of nodes that experience a shock or who first hear about a new product.

Empirical tests of the relationship between aggregate network-level outcomes and network statistics involve estimating specifications such as Eq. 17. The shape of the function $\boldsymbol {f}_{\bar {\boldsymbol {y}}}(.)$ and the choice of statistics in $\bar {\boldsymbol {w}}_{\bar {\boldsymbol {y}}}(\boldsymbol {G})=\bar {\boldsymbol {\omega }}$, where $\bar {\boldsymbol {\omega }}$ is an $(M\times \bar {R})$ matrix of network statistics, are again, ideally, motivated by theory. With linear $\boldsymbol {f}_{\bar {\boldsymbol {y}}}(.)$, this implies the following equation:

20 $$ \bar{\boldsymbol{Y}}=\phi_{0}+\bar{\boldsymbol{\omega}}\boldsymbol{\phi}_{1}+\bar{\boldsymbol{X}}\boldsymbol{\phi}_{2}+\bar{\boldsymbol{w}}_{\bar{\boldsymbol{x}}}(\boldsymbol{G},\,\bar{\boldsymbol{X}})\boldsymbol{\phi}_{3}+\boldsymbol{u}  $$

where the variables are as defined after Eq. 17. The parameter of interest is typically ***ϕ***_1_. Defining $\bar {\boldsymbol {W}}=\left (\boldsymbol {\omega },\bar {\boldsymbol {X}},\bar {\boldsymbol {w}}_{\bar {\boldsymbol {x}}}\left (\boldsymbol {G},\,\bar {\boldsymbol {X}}\right)\right)$, the key identification assumption is that $\mathrm {E}\left [\boldsymbol {u}\bar {\boldsymbol {W}}\right ]=0$, which will not hold if there are unobserved variables in ***u*** that affect both the formation of the network and the outcome $\bar {\boldsymbol {y}}$; or if the network statistics are mismeasured. Recent empirical work, such as that by Banerjee et al. (2013), has used quasi-experimental variation to try and alleviate some of the challenges posed by the former issue in identifying the parameter ***ϕ***_***1***_.

Since this specification uses data at the network level, estimation will require a large sample of networks in order to recover precise estimates of the parameters, even in the absence of endogeneity from network formation and mismeasurement of the network. This is a problem in practice, since networks data are difficult and costly to collect, meaning that in practice researchers have data for a small number of networks only.

## Experimental variation

Thus far, we have considered the identification of the social effect parameters using observational data. We now consider identification of these parameters using experimental data. We focus on the case where a policy is assigned randomly to a sub-set of nodes in a network. Throughout, we assume that the network is pre-determined and unchanged by the exogenously assigned policy^23^.

We focus the discussion on identifying parameters of the local average model specified in the “Local average models” section above, studied by Dieye et al. (2014). The issues related to using experimental variation to uncover the parameters of the local aggregate model are similar. As outlined above, this model implies that a node’s outcome is affected by the average outcome of its network neighbours, its own and network-level exogenous characteristics (the latter may be subsumed into a network fixed effect), and the average characteristics of its network neighbours. We are typically interested in parameters *β*, ***γ*** and ***δ*** in the following equation:

21 $$ \boldsymbol{Y}=\alpha\boldsymbol{\iota}\mathbf{+\,}\beta\tilde{\boldsymbol{G}}\boldsymbol{Y}+\boldsymbol{X}{\boldsymbol{\gamma}} +\tilde{\boldsymbol{G}}\boldsymbol{X}{\boldsymbol{\delta}}+\boldsymbol{L}\tilde{\boldsymbol{\nu}}+{\boldsymbol{\varepsilon}}   $$

where the variables are as defined previously.

Throughout this section, we assume that the policy shifts outcomes for the nodes that directly receive the policy^24^. To proceed further, we first assume that a node that does not receive the policy (i.e. is untreated, to use the terminology from the policy evaluation literature) is only affected by the policy through its effects on the outcomes of the node’s network neighbours. This implies the following model for the outcome ***Y***:

22 $$ \boldsymbol{Y}=\alpha\boldsymbol{\iota}\mathbf{+\,}\beta\tilde{\boldsymbol{G}}\boldsymbol{Y}+\boldsymbol{X}{\boldsymbol{\gamma}}+\tilde{\boldsymbol{G}}\boldsymbol{X}{\boldsymbol{\delta}}+\rho\boldsymbol{t}+\boldsymbol{L}\tilde{\boldsymbol{\nu}}+{\boldsymbol{\varepsilon}}  $$

where ***t*** is the treatment vector and *ρ* is the direct effect of treatment. We assume that $\mathbb {E}\big [\boldsymbol {\varepsilon }|\boldsymbol {X},\boldsymbol {Z},\tilde {\boldsymbol {G}},\boldsymbol {t}\big ]=0$. Moreover, random allocation of the treatment implies that . Applying the same within-transformation as in the ‘Local average models’ section above to account for the network-level fixed effect leads to the following specification:

23 $$ \boldsymbol{J}\boldsymbol{Y}=\alpha\boldsymbol{J}\boldsymbol{\iota}\mathbf{+\,}\beta\boldsymbol{J}\tilde{\boldsymbol{G}}\boldsymbol{Y}+\boldsymbol{J}\boldsymbol{X}{\boldsymbol{\gamma}}+\boldsymbol{J}\tilde{\boldsymbol{G}}\boldsymbol{X}{\boldsymbol{\delta}}+\rho\boldsymbol{J}\boldsymbol{t}+\boldsymbol{J}{\boldsymbol{\varepsilon}}  $$

We can use instrumental variables to identify *β* as long as the deterministic part of the right-hand side of Eq. 23, $\big [\mathrm {E}(\boldsymbol {J}\tilde {\boldsymbol {G}}\boldsymbol {Y}),\boldsymbol {J}\boldsymbol {X},\boldsymbol {J}\tilde {\boldsymbol {G}}\boldsymbol {X},\boldsymbol {J}\boldsymbol {t}\big ]$ has full column rank. ***JX*** and $\boldsymbol {J}\tilde {\boldsymbol {G}}\boldsymbol {X}$ can be used as instruments for themselves. We thus need an instrument for $\mathbb {E}\big [\boldsymbol {J}\tilde {\boldsymbol {G}}\boldsymbol {Y}\big ]$. We use the following expression for $\boldsymbol {J}\tilde {\boldsymbol {G}}\boldsymbol {Y}$, derived from the reduced form of Eq. 21 under the assumption that |*β*|<1, to construct instruments:

24 $$\begin{array}{*{20}l} \mathbb{E}\left[\boldsymbol{J}\tilde{\boldsymbol{G}}\boldsymbol{Y}\right]=&\boldsymbol{J}\tilde{\boldsymbol{G}}{\sum_{s=0}^{\infty}\beta^{s}\tilde{\boldsymbol{G}^{s}}}\alpha\boldsymbol{\iota}+\boldsymbol{J}\left(\tilde{\boldsymbol{G}}\boldsymbol{X}\boldsymbol{\gamma}+\beta\tilde{\boldsymbol{G}^{2}}\boldsymbol{X}\boldsymbol{\gamma}+\ldots\right)\\&+\boldsymbol{J}\left(\tilde{\boldsymbol{G}^{2}}\boldsymbol{X}\boldsymbol{\delta}+\beta\tilde{\boldsymbol{G}^{3}}\boldsymbol{X}\boldsymbol{\delta}+\ldots\right) \\ &+\boldsymbol{J}\left(\rho\tilde{\boldsymbol{G}}\boldsymbol{t}+\beta\rho\tilde{\boldsymbol{G}^{2}}t+\ldots\right) \end{array} $$

From this equation, we can see that $\tilde {\boldsymbol {G}}\boldsymbol {t}$, the average treatment status of a node’s network neighbours, appears in the reduced form for $\mathbb {E}[\boldsymbol {J}\tilde {\boldsymbol {G}}\boldsymbol {Y}]$. However, it does not appear in Eq. 22. It can thus be used as an instrument for $\tilde {\boldsymbol {G}}\boldsymbol {Y}$, either in addition to, or as an alternative to $\tilde {\boldsymbol {G}}^{2}\boldsymbol {X}$ and $\tilde {\boldsymbol {G}}^{3}\boldsymbol {X}$, the average characteristics of the node’s second- and third-degree neighbours. Thus, the policy could be used to identify the model parameters, albeit under a strong assumption on the mechanism by which it has any effect (see below)^25^.

An advantage to using $\tilde {\boldsymbol {G}}\boldsymbol {t}$ as an instrument for the endogenous $\tilde {\boldsymbol {G}}\boldsymbol {Y}$ is that, since the treatment is randomly assigned, and if it only directly affects the treated individual’s outcome, it might be a more plausible instrument than $\tilde {\boldsymbol {G}}^{2}\boldsymbol {X}$ and $\tilde {\boldsymbol {G}}^{3}\boldsymbol {X}$. A second advantage of this instrument relative to $\tilde {\boldsymbol {G}}^{2}\boldsymbol {X}$ and $\tilde {\boldsymbol {G}}^{3}\boldsymbol {X}$ is that it only requires knowledge of agents’ direct neighbours. The identification conditions outlined previously in the “Local average models” to “Hybrid local models” sections hinge on the fact that within the network of interest, some agents are only indirectly connected, which might be too strong in some contexts. Identification here requires sufficient variation in the proportion of an agents’ direct neighbours that are treated for it to be a powerful instrument, which imposes fewer restrictions on network structure (e.g. there should be variation in degree within the network). As a result, the social effect parameter can be identified in a wider range of network structures.

In many cases, however, the assumption that the policy affects a node’s outcome only if it is directly treated may be too strong. The treatment status of a node’s neighbours could affect its outcome even when the neighbours’ outcomes do not shift in response to receiving the policy. An example of such a case, studied by Banerjee et al. (2013), is when the treatment involves providing individuals with information on a new product, and the outcome of interest is the take-up of the product. Then neighbours’ treatment status could affect the individual’s own adoption decision by (1) shifting his neighbours’ decision (endorsement effects) and also (2) through neighbours passing on information about the product and letting the individual know of its existence (diffusion effect)^26^. In this case, which is studied by Dieye et al. (2014), a more appropriate model would be as follows:

25 $$ \boldsymbol{Y}=\alpha{\boldsymbol{\iota}}+\beta\tilde{\boldsymbol{G}}\boldsymbol{Y}+\boldsymbol{X}\boldsymbol{\gamma}+\tilde{\boldsymbol{G}}\boldsymbol{X}\boldsymbol{\delta}+\rho\boldsymbol{t}+\tilde{\boldsymbol{G}}\boldsymbol{t}\mu+\boldsymbol{\varepsilon}  $$

where *ρ* captures the direct treatment effect, i.e. the effect of a node itself being treated, and *μ* is the direct effect of the average treatment status of social contacts. This highlights the limits to using exogenous variation from randomised experiments to identify social effect parameters. We might want to use the exogenous variation in the average treatment allocation of a node’s neighbours, $\tilde {\boldsymbol {G}}\boldsymbol {t}$, as an instrument for neighbours’ outcomes, $\tilde {\boldsymbol {G}}\boldsymbol {Y}$. However, this will identify *β* only under the assumption that *μ*=0, i.e. there is no direct effect of neighbours’ treatment status. This rules out economic effects such as the diffusion effect.

We can still make use of the treatment effect for identification, by using the average treatment status of a node’s second-degree (and higher-degree) neighbours, $\tilde {\boldsymbol {G}^{2}}\boldsymbol {t}$, as instruments for the average outcome of his neighbours ($\tilde {\boldsymbol {G}}\boldsymbol {Y}$). This is the same identification result as discussed earlier, from Bramoullé et al. (2009), and simply treats $\tilde {\boldsymbol {G}^{2}}\boldsymbol {t}$ in the same way the other covariates of second-degree neighbours, $\tilde {\boldsymbol {G}^{2}}\boldsymbol {X}$. Such instruments rely not only on variation in treatment status, but also on the network structure, with identification not possible for certain network structures as we saw in the “Local average models” section. As before, note that instruments based on random treatment allocation and network structure (e.g. $\tilde {\boldsymbol {G}}\boldsymbol {t}$ and $\tilde {\boldsymbol {G}^{2}}\boldsymbol {t}$) will be more plausible than those based on the exogenous characteristics, ***X***, and the network structure (e.g. $\tilde {\boldsymbol {G}}^{2}\boldsymbol {X}$) since ***t*** has been randomly allocated, whereas ***X*** need not be^27^.

Thus far, we have discussed how exogenous variation arising from the random assignment of a policy can be used to identify the social effect associated with a specific model—the local average model—which, as we saw, arises from an economic model where agents conform to their peers. In empirical work, though, it is common for researchers to directly include the average treatment status of network neighbours, rather than their average outcome, as a regressor in the model. In other words, the following type of specification is usually estimated:

26 $$ \boldsymbol{Y}=b_{1}\boldsymbol{\iota}+b_{2}\tilde{\boldsymbol{G}}\boldsymbol{t}+\boldsymbol{X}\boldsymbol{b}_{3}+\tilde{\boldsymbol{G}}\boldsymbol{X}\boldsymbol{b}_{4}+b_{5}\boldsymbol{t}+\boldsymbol{u}  $$

A non-zero value for *b*_2_ is taken to indicate the presence of some social effect. However, without further modelling, it is not possible to shed light on the exact mechanism underlying this social effect, or the value of some ‘deep’ structural parameter.

When within-network experimental variation is used to identify social effects, careful attention must be paid to the important issue of inference. At one extreme, a researcher might have many (often thousands) of nodes embedded in the same network, and construct a valid comparison group using similar nodes embedded in a different part of the network to the treated node. The complication in computing standard errors comes from the fact that all nodes are embedded within the same network, and may face correlated unobserved shocks. Though these shocks may not affect identification, they will generate correlations in the outcomes of units, and must be accounted for when conducting inference. The availability of only a single network makes it very difficult to derive large sample approximations of distributions and thereby to calculate valid standard errors. Athey et al. (2015) extend the method of randomisation inference, which calculates exact *p* values, to this setting. Under randomisation inference, the distribution of the test statistic is generated by considering all possible realisations of the treatment assignment, keeping the potential outcomes and characteristics of units fixed. A drawback of this procedure is that it allows for testing of sharp null hypotheses—e.g. the treatment has no effect whatsoever—only. However, we often want to test non-sharp hypotheses. Athey et al. (2015) develop methods for the computation of *p* values for three specific null hypotheses.

## Conclusions

In this paper, we provide an overview of methods to identify social effects in linear social effect models using a single cross section of data. For a number of the most commonly used specifications of linear local models, we provide an overview of the theory models that could generate the empirical linear local specification, before outlining the conditions under which the social effect parameters of interest are identified. Thereafter, we describe what is known so far about non-local models, before considering how experimental and quasi-experimental variation can be used to identify the social effects. Our focus is on methods that take the network to be (conditionally) exogenous as well as perfectly observed by the researchers.

When data are available only on agents and the reference groups to which they belong, researchers have for some time worried about how social effects might be identified. However, when detailed data on nodes and their individual links are present, identification of social effects (taking the network as conditionally exogenous) is generic, and estimation is relatively straightforward. Three broader conceptual issues exist in this case.

First, theory is often silent on the precise form that peer effects should take when they exist. Since Manski (1993), many people have focused on the ‘local average’ framework, often without discussion of the implications for economic behaviour, but social effects might instead take a local aggregate, or indeed local maximum/minimum form where the best child in a classroom provides a good example to all others, or the worst disrupts the lesson. Until a non-parametric way of allowing for social effects is developed, researchers need to use theory to guide the empirical specification they use.

Second, researchers typically treat the observed network as the network which mediates the social effect, and where many networks are observed the union of these is taken. However, this could generate important biases if the actual network is different to that observed, either due to measurement error or because a different type of relationship is the relevant one for mediating a particular effect. Here again it is important that some justification is given for why the network used should be the appropriate one.

Finally, the observed network is typically treated as exogenous, or at least the errors are treated as being mean independent of the network. In many circumstances, one might imagine that agents choose which links to form, and these choices may depend on characteristics that are neither observed by the econometrician nor independent of the outcome. This creates a problem for identification strategies that rely on the absence of links (i.e. using friends-of-friends), since the absence of a link may contain some information about the difference in the unobservables. For instance, more motivated pupils in a school may choose to link with other motivated pupils, or individuals may choose to become friends with other individuals who share a common interest (such as an interest in reading, or mathematics) that is unobserved in the data available to the researcher. In such examples, the absence of a link is due to the unobserved terms of the two agents being correlated in a specific way rather than the absence of correlation between these terms. Recent literature, including Goldsmith-Pinkham and Imbens (2013), Blume et al. (2015), Arduini et al. (2015) and Horace et al. (2015) among many others, has begun considering solutions to this issue. Many of these methods are reviewed in a longer working paper version of this article (see Advani and Malde 2014).

Much work has been done to develop methods for working with network data, both in economics and in other fields. Applied researchers can therefore take some comfort in knowing that many of the challenges they face using these data are ones that have been considered before, and for which there are typically at least partial solutions already available. While the limitations of currently available techniques mean that empirical results should be interpreted with some caution, attempting to account for social effects is likely to be less restrictive than simply imposing that they cannot exist.

## Footnotes

1 Brock and Durlauf (2001); Brock and Durlauf (2007) study identification of social effects in models with discrete outcomes.

2 Panel data on networks are becoming increasingly available. Such longitudinal information can be useful in making identification more credible by, for example, allowing for models that account for fixed unobserved variables that affect both the network and the outcomes of interest. Studies making use of such data to estimate peer effects include Patnam (2013); Goldsmith-Pinkham and Imbens (2013); Comola and Prina (2014). The former studies use the panel data to account for network endogeneity. More commonly, studies often have available data with a panel dimension of outcomes, but only a single measurement of the network. The methods outlined in this paper can be applied in these cases, though stronger restrictions could also be imposed improving the credibility of identification.

3 It is possible to overcome the reflection problem without detailed networks data. Possible methods include using conditional variance restrictions as in Graham (2008), or using variation in network/group size as in Lee (2007). Blume et al. (2010) provide a more complete overview of the different methods.

4 See Sacerdote (2011) for an overview of this literature.

5 In a slight abuse of notation, we will also use *g* to index individual networks when data from multiple networks are available.

6 $\boldsymbol {G}_{i,g}^{\prime }$ is the *i*^th^ row of $\boldsymbol {G}_{g}^{\prime }$, which is the *i*^th^ column of ***G***_*g*_.

7 A row stochastic (also called ‘right stochastic’ matrix) is one whose rows are normalised so they each sum to one.

8 Identification of parameters in such a model was first systematically considered by Manski (1993).

9 When data on only a single network are available, the empirical specification is as follows: $\boldsymbol {y}_{g}=\tilde {\nu }_{g}\boldsymbol {\iota }+\beta \tilde {\boldsymbol {G}}_{g}\boldsymbol {y}_{g}+\boldsymbol {X}_{g}{\boldsymbol {\gamma }+}\tilde {\boldsymbol {G}_{g}}\boldsymbol {X}_{g}\boldsymbol {\delta +}\boldsymbol {\varepsilon }_{g}$, where $\tilde {\nu _{g}}=\alpha +\boldsymbol {z}_{g}\boldsymbol {\eta }+\nu _{g}$ in our earlier notation, capturing all of the network-level characteristics.

10 Notice that in Eq. 2, $\sum _{j=1}^{N_{g}}\tilde {G}_{ij,g}y_{j,g}$ is identical to the *i*^*t**h*^ row of $\tilde {\boldsymbol {G}_{g}}\boldsymbol {y}_{g}$, which appears in Eq. 1.

11 Consumer demand models where, given prices, an individual’s demand increases with average demand of some reference group also generate a similar specification (see Gaertner 1974; Pollak 1976; Alessie and Kapteyn 1991; Case 1991).

12 Similar identification results have been independently described by De Giorgi et al. (2010), who have data with overlapping peer groups of students who share a number of classes.

13 Equivalently, a triple such ,  and  forms an intransitive triad.

14 This definition is also referred to as the clustering coefficient.

15 The games considered in both Bramoulle and Kranton (2007) and Bramoullé et al. (2014) are not strictly linear models, since there are corner solutions at zero.

16 Notice that $\sum _{j=1}^{N_{g}}G_{ij,g}y_{j,g}=\boldsymbol {G}_{i,g}\boldsymbol {y}_{g}$.

17 A more general definition for Katz-Bonacich centrality is ***b***(***G***_*g*_,*β*,*a*)=(***I***_*g*_−*β****G***_*g*_)^−1^(*a****G***_*g*_***ι***_*g*_), where *a*>0 is a constant (Jackson 2008).

18 The condition that |*β**ω*_*max*_(***G***_*g*_)|<1 ensures that positive feedback loops set off by these complementarities don’t diverge without bound. The extent to which it binds will depend on the underlying network topology: the largest eigenvalue increases with additional links so that in larger and denser networks the largest possible value of *β* for which the condition holds will be smaller.

19 See Liu et al. (2012) for a different identification condition that allows for some linear dependence among these matrices under additional restrictions.

20 With additional restrictions, identification can still be achieved when there is some linear dependence in these matrices. See Liu et al. (2012) for details.

21 As with the local average and local aggregate model, this identification assumption imposes that there are pairs of individuals who influence the other (or each other) indirectly only, which might be very strong in some contexts.

22 The term ***w***_***y***_(***G***, ***Y***) will be endogenous when network statistics are interacted with ***Y***.

23 This assumption is not innocuous. Comola and Prina (2014) provide an example where the policy intervention does change the network.

24 Below, we will consider identification conditions in the case where a node may be affected by the treatment status of his network neighbours even if their outcomes do not shift in response to the treatment.

25 Similar results can be shown for the local aggregate model when |*β**ω*_*max*_(***G***)|<1. However, as shown above, node degree can also be used as an additional instrument in this model.

26 The study of how to use these effects to maximise the number of people who adopt relates closely to study of the ‘key player’ in work by Ballester et al. (2006) and Liu et al. (2012).

27 However, it is important to remain aware that if some variables in ***X*** are not exogenous (and so not suitable instruments when combined with network matrices), then their inclusion in estimation equation will already be a problem.
